# S255. METAPHORICAL CONCEPTUALIZATION OF SCHIZOPHRENIA AND THE CAREGIVING PROCESS FROM THE PERSPECTIVE OF PRIMARY FAMILY CAREGIVERS

**DOI:** 10.1093/schbul/sby018.1042

**Published:** 2018-04-01

**Authors:** Zhou De-Hui Ruth, Chiu Yu-Lung Marcus, Lo Tak-Lam William, Lo Wai Fan Alison, Wong Siu Sing

**Affiliations:** 1 Hong Kong Shue Yan University; 2 City University of Hong Kong; 3 Kwai Chung Hospital

## Abstract

**Background:**

According to Hong Kong Hospital Authority (2011), nearly 20,000 outpatients with schizophrenia in Hong Kong demand substantial family support. The family caregivers are the individuals who take care of persons with schizophrenia daily, check their medication and provide emotional support. They are enlisted as important therapeutic agents. How family caregivers understand and perceive schizophrenia and the caregiving process will influence not only the quality and their persistence of the caregiving, but also the rehabilitation process of persons with schizophrenia. Thus, it is important to investigate their conceptualization of schizophrenia and the caregiving process to inform schizophrenia mental health family recovery work.

**Methods:**

This study used a mixed method of quantitative and qualitative design. In the quantitative part, a questionnaire about the metaphors for schizophrenia and the caregiving process were administrated to 194 caregivers whose family members were diagnosed with schizophrenia according to the 2016 version of ICD-10-CM Diagnosis Code. This questionnaire also included standardized instruments, such as Brief Family Relationship Scale (BFRS), Experience of Caregiving Inventory (ECI-66), Mental Health Inventory(MHI-5) and Inner Resource Scale (SAS-I). Among the participants, 147 were women caregivers and 47 were male caregivers. In the qualitative part, a focus group interview with 8 randomly selected caregiver participants were invited to talk about their caregiving experiences and their understanding of schizophrenia.

**Results:**

The dominant metaphors for schizophrenia were reported as unexpected visitors, the Anakin Skywalker, a time bomb, and a fire alarm. These metaphors vividly describe the unexpectedness of episodes that schizophrenia outpatients experienced as well as the explosive damages that schizophrenia caused their families. 73.2% participants used “climbing up the mountain” to describe their caregiver experiences and emphasize on the necessity of overcoming difficulties during the caregiving process. 67.5% caregivers preferred to use the “rescue work of firefighters” to describe the nature of their caregiving. 50% participants indicated the caregiving work as a burden that they chose not to lay down no matter how heavy it is, whereas 17.5% caregivers regarded it as a burden that they cannot shake off and always restricts their freedom. The independent t-tests showed that adult children caregivers reported statistically significantly more positive personal experiences t (94) = -2.423, p < .05, d = .26, readiness to seek for information t (94) = -2.860, p < .01, d = .61, and positive communication t(94) = -2.625, p < .01, d = .56 than spouse caregivers.

**Discussion:**

The metaphorical conceptualization for schizophrenia and the meaning of the caregiving process from the caregivers’ perspectives have strong implications for schizophrenia family recovery work. They could help to frame psychotherapy sessions for caregiver group works. According to the narrative psychotherapy approach, the metaphor of “unexpected visitors” could help caregivers to externalize the problem of schizophrenia and gain a space to describe and deal with schizophrenia in alliance with their family members.

**Acknowledgement:**

The reported study was funded by the Research Grant Council of HKSAR (UGC/FDS15/M01/15). However, the opinions expressed do not necessarily reflect the positions of the funding agency. We want to acknowledge our great gratitude to Kwai Chung hospital for their support in multiple aspects and all caregiver participants for their active participation in our research.

